# Chunking as a function of sequence length

**DOI:** 10.1007/s10071-024-01835-z

**Published:** 2024-03-02

**Authors:** Laure Tosatto, Joël Fagot, Dezso Nemeth, Arnaud Rey

**Affiliations:** 1 Aix Marseille Univ, CNRS, LPC, Marseille, France; 2 Aix Marseille Univ, ILCB, Aix-en-Provence, France; 3 Station de Primatologie Celphedia, CNRS, Rousset, France; 4 INSERM, Université Claude Bernard Lyon 1, CNRS, Centre de Recherche en Neurosciences de Lyon CRNL U1028 UMR5292, Bron, France; 5 NAP Research Group, Institute of Psychology, Eötvös Loránd University & Institute of Cognitive Neuroscience and Psychology, HUN-REN Research Centre for Natural Sciences, Budapest, Hungary; 6 Department of Education and Psychology, Faculty of Social Sciences, University of Atlántico Medio, Las Palmas de Gran Canaria, Spain; 7 Normandie Univ, UNICAEN, CNRS, ETHOS, 14000 Caen, France; 8 Present Address: Aix Marseille Univ, CNRS, CRPN, Marseille, France

**Keywords:** Chunking, Sequence learning, Non-human primates, Associative learning

## Abstract

Chunking mechanisms are central to several cognitive processes. During the acquisition of visuo-motor sequences, it is commonly reported that these sequences are segmented into chunks leading to more fluid, rapid, and accurate performances. The question of a chunk’s storage capacity has been often investigated but little is known about the dynamics of chunk size evolution relative to sequence length. In two experiments, we studied the dynamics and the evolution of a sequence’s chunking pattern as a function of sequence length in a non-human primate species (Guinea baboons, *Papio papio*). Using an operant conditioning device, baboons had to point on a touch screen to a moving target. In Experiment 1, they had to produce repeatedly the same sequence of 4 movements during 2000 trials. In Experiment 2, the sequence was composed of 5 movements and was repeated 4000 times. For both lengths, baboons initially produced small chunks that became fewer and longer with practice. Moreover, the dynamics and the evolution of the chunking pattern varied as a function of sequence length. Finally, with extended practice (i.e., more than 2000 trials), we observed that the mean chunk size reached a plateau indicating that there are fundamental limits to chunking processes that also depend on sequence length. These data therefore provide new empirical evidence for understanding the general properties of chunking mechanisms in sequence learning.

**Supplementary Information:**

The online version contains supplementary material available at 10.1007/s10071-024-01835-z.

## Introduction

A key mechanism allowing our cognitive system to compress information and increase short term memory capacity is the formation of chunks (Mathy and Feldman 2012; Miller 1956). Chunking is defined as the process of associating and grouping several items together into a single processing unit (Gobet et al. 2001, 2016). Several studies have questioned the maximum number of chunks that can be stored in short-term memory. While Miller (1956) initially proposed that humans have a short-term storage capacity of 7 plus or minus 2 chunks, Cowan (2001) suggested that this capacity might be more limited to a set of approximately four chunks. Other studies were concerned by the number of items that can be stored into a single chunk and have shown that chunks seem to have their own limits regarding storage, seemingly 3 or 4 items per chunk (Allen and Coyne 1988; Chase and Simon 1973; Johnson 1970). Yet, this absolute number of chunk size varies depending on experimental paradigms and factors such as expertise. For example, Gobet and Clarkson (2004) found that chess Masters were able to chunk many pieces of information (up to 15 items). Here, we aim to investigate the size of chunks and their evolution during sequence learning and the effect of sequence length on the chunking pattern.

In the field of perceptual-motor learning, chunking has been considered as the main motor sequence integration mechanism (Diedrichsen and Kornysheva 2015; Wymbs et al. 2012). Motor sequence learning is commonly described as the process by which a sequence of movements is acquired and executed with increased speed and accuracy (Willingham 1998). This process is largely related to the question of chunking as individuals spontaneously parse sequences of movements into chunks corresponding to subparts of the sequence. This process of parsing into chunks becomes clear when studying the pattern of successive response times (RTs) in typical sequential button-press tasks: long temporal gaps between two successive responses are usually observed and are assumed to mark chunk boundaries (Abrahamse et al. 2013; Bottary et al. 2016). The resulting chunking pattern therefore reflects the sequence’s organization in memory (Sakai et al. 2003) and inform us about the length chunks can have.

If many studies report that chunks typically contain 3 or 4 items, sometimes 5 (e.g., Nissen and Bullemer 1987; Sakai et al. 2003; Verwey 1996; Verwey et al. 2002), other studies found much larger chunk sizes of 7 or 8 (e.g., Kennerley et al. 2004). One factor that can explain this heterogeneity of results is practice. Indeed, some studies include a very limited number of repetitions of the same sequence (e.g., only 36 repetitions in Rosenbaum et al. 1983) whereas others are interested in extended practice and include hundreds of trials (e.g., 588 in Verwey 2003). Throughout extended practice, chunks were found to evolve and grow larger as if more compression of information was possible with increasing familiarity with the sequence (e.g., Acuna et al. 2014; Bera et al. 2021; Ramkumar et al. 2016; Wright et al. 2010). These conclusions are not limited to humans and identical results have been obtained in other animals, particularly non-human-primates (e.g., Ramkumar et al. 2016; Terrace 2002; Scarf et al. 2018). Animals too appear to spontaneously chunk sequences and the chunking pattern can evolve through extended practice.

Another factor that may influence chunk size is the length of the sequence. Indeed, temporal gaps between items of the sequence seem to emerge only after sequences of 3 or 4 items (Bo et al. 2009; Verwey and Eikelboom 2003). This suggests that a single chunk can be formed for very short sequences and that as the sequence gets longer, more chunks can emerge. For instance, Verwey (2003) found no segmentation in 2 and 4-item sequences whereas chunking occurred when participants performed 6-item sequences. This experiment however does not specifically study the evolution of chunk sizes in relationship to sequence length.

In a recent study, Tosatto et al. (2022) studied the evolution of chunks during the repeated execution of a single visuo-motor sequence in non-human primates (i.e., Guinea baboons *papio papio*). Using a serial response time (SRT) task, baboons had to repeatedly produce the same sequence composed of 9 different locations for a thousand trials. Consistent with previous studies, results showed that baboons initially parsed the sequence into small chunks that progressively became fewer and longer throughout the task. Indeed, the average chunk size was initially equal to 2.2 items per chunk and it increased up to 3.38 items per chunk at the end of the experiment, after extended practice. On some occasions, longer chunks of 8 or 9 items were also observed.

This experiment also showed that the evolution of the chunks was governed by two reorganization mechanisms: *concatenation* (i.e., the process by which two successive chunks are performed more fluidly and the temporal gap between them decreases leading to a single and longer chunk) and *recombination* (i.e., the emergence of a new segmentation pattern across chunks, such as two chunks of 3 items become a chunk of 4 items followed by a chunk of 2 items). Tosatto et al.’s (2022) study therefore informs us about the relative flexibility of chunks throughout learning, but this study remains limited because using only 9-items sequences does not provide information about the relationship between the initial chunking pattern, its evolution and the length of the sequence.

The aim of the present research is to study the dynamics of chunking for shorter extensively repeated sequences, in comparison to the results obtained for 9-item sequences. We designed two experiments to study the evolution of chunk size for different sequence lengths. In the first experiment, baboons were trained on a single repeated sequence of 4 items for 2000 trials. This specific sequence length was chosen as it is generally accepted in the literature that chunks can store up to 3 or 4 items. Therefore, we expected either no segmentation in producing the sequence, or an initial segmentation of the sequence followed by a progressive increase in chunk size up to 4 items. The second experiment was similar to the first, but baboons were trained on a 5-item sequence for 4000 trials. This larger sequence length was used as a proxy to infer the evolution of chunk size for a sequence length between 9 and 4 items, using the data already collected for these two latter lengths. We also increased the total number of trials to determine if greater extended practice would still lead to a linear increase of the average chunk size.

## Experiment 1

### Method

#### Participants

Twenty-five Guinea baboons (*Papio papio*) from the CNRS primate facility in Rousset (France) were tested in this study. For practical reasons, we stopped the experiment after 17 monkeys completed all scheduled trials (fourteen female and three male, age range 2.8–24.8 years). Water was provided ad libitum during the test, and the monkeys received their normal food ration of fruits every day at 5 PM.

#### Materials

##### Apparatus

This experiment was conducted with a computer-learning device based on the voluntary participation of baboons (for details, see Fagot and Bonté 2010). Baboons implanted with a RFID microchip had free access to 10 automatic operant conditioning learning devices equipped with touch screens. Each time a monkey entered a test chamber, it was identified by its microchip and the system resumed the trial list where the subject left it at its previous visit. The experiment was controlled by E-prime (Version 2.0, Psychology Software Tools, Pittsburgh, PA, USA).

##### Task and stimuli

**Fig. 1:**
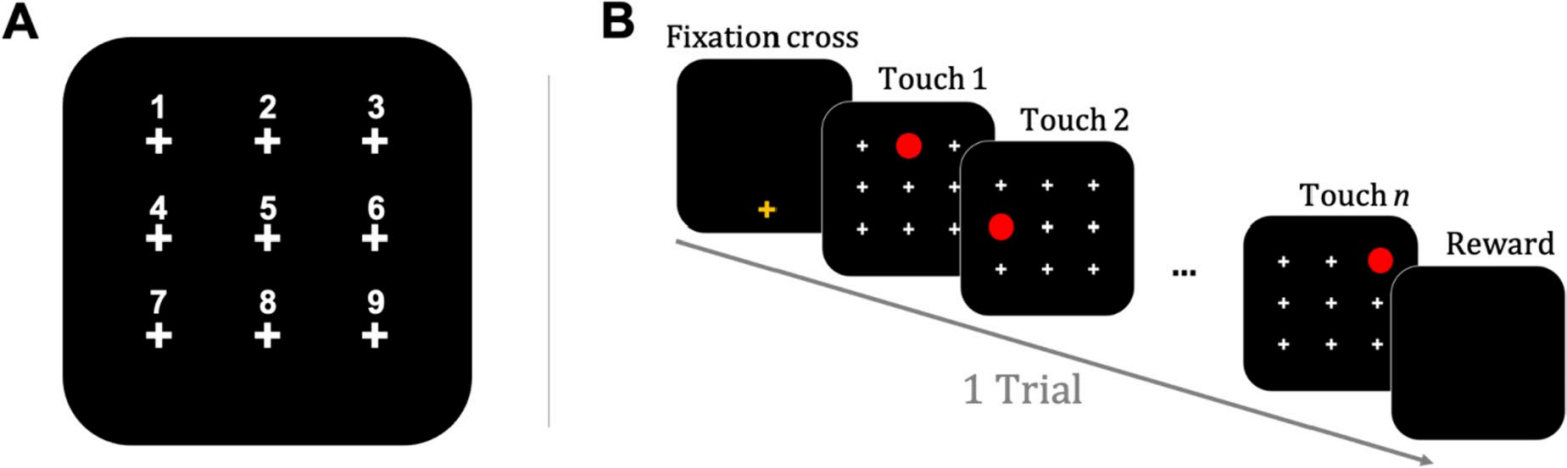
Experimental display and stimulus presentation.** A** Display of the 9 uniformly spaced predetermined locations (white crosses) virtually labeled as position 1–9 (i.e., only the white crosses were displayed, not the numbers). **B** Example of a single trial (ISI = 0)

The screen was divided into nine uniformly spaced predetermined locations represented by white crosses on a black background, virtually labeled as Position 1 to 9 (see Fig. 1A). A trial began with the presentation of a yellow fixation cross at the bottom of the screen. Once pressed, the fixation cross disappeared and the nine white crosses were displayed, one of them being replaced by the target, a red circle. When the target was touched, it was immediately replaced by the cross. The red circle then replaced the next position in the sequence until it was touched, and a new position was displayed. Reward (grains of dry wheat) was provided at the end of a sequence of four touches (see Fig. 1B).

If baboons touched an inappropriate location (incorrect trial) or failed to touch the screen within 5000 ms after the red circle’s appearance (aborted trial), a green screen was displayed for 3000 ms as a marker of failure. Aborted trials were not retained and therefore presented again, while incorrect trials were not. The time elapsed between the appearance of the red circle and the baboon’s touch on this circle was recorded as the response time (RT) for that location in the sequence. To learn the task, baboons initially received random trials that were rewarded after three touches. Then, the number of touches in a trial was increased to four.

##### Design of the sequences

To control the motor difficulty of the transitions to be produced in the sequence, a random phase of sequence production was first conducted, where thirteen baboons performed random sequences of six positions for 1000 trials. For each of these 13 baboons, we computed all the mean transition times from one location to another, leading to a 9 × 9 matrix of RTs (with no values on the diagonal of the matrix). We then correlated each matrix of each baboon to the matrix of all the other baboons and, on average, the correlation between these matrices was 0.42 (SD = 0.19), indicating that there was a good consistency between the baboons’ performances. This result allowed us to compute an average baseline measure for all possible transitions for the entire group of baboons (see Appendix 1).

Based on these baseline measures, we designed four sequences of four serial positions for which each transition T was numerically faster (or equally fast) to produce on average than the next one (i.e., T1 ≤ T2 ≤ T3). Ideally, all the transitions should be matched to equate each transition for motor difficulty and to study the segmentation/chunking of the sequence. This was not possible for sequences of 9 positions (i.e., in Tosatto et al. 2022) and that is why we constructed the repeated sequences by systematically choosing increasing or equal transition times from the first transition to the last. Therefore, to make this study (with sequences of 9 positions) comparable with the present study with shorter sequences, we adopted the same logic when constructing the sequences. However, we also made sure that there was no significant difference between all the transition times of each sequence so that at the beginning of learning there was no significant difference on any transition (see Supplementary Materials). Appendix 2 provides the details of all the sequences we used, i.e., the sequence itself, the average response times for each transition and the number of monkeys that were presented with each sequence.

#### Procedure

To neutralize the potential effect of one specific sequence, baboons were exclusively presented with either Sequence 1 (n = 4), 2 (n = 5), 3 (n = 3) or 4 (n = 5) and had to produce their sequence repeatedly for 2000 successive trials. RTs for each position of the sequence were recorded for all the trials.

### Results

On average, baboons required 2.82 days (*SD* = 1.19) to complete the 2000 trials, with a mean of 708.33 trials per day and a mean accuracy level of 99.44% (*SD* = 1.65). Incorrect trials were removed from the dataset (0.56%). RTs greater than 1000 ms were excluded and an additional recursive trimming procedure excluded RTs greater or smaller than 2.5 standard deviations from the subject’s mean for each of the four possible positions (15%). Note that by not removing any outlier, this does not change the main trends of our results (see Supplementary Materials). RTs for each of the four positions and for the 2000 trials were divided into 20 Blocks of 100 trials.

**Fig. 2:**
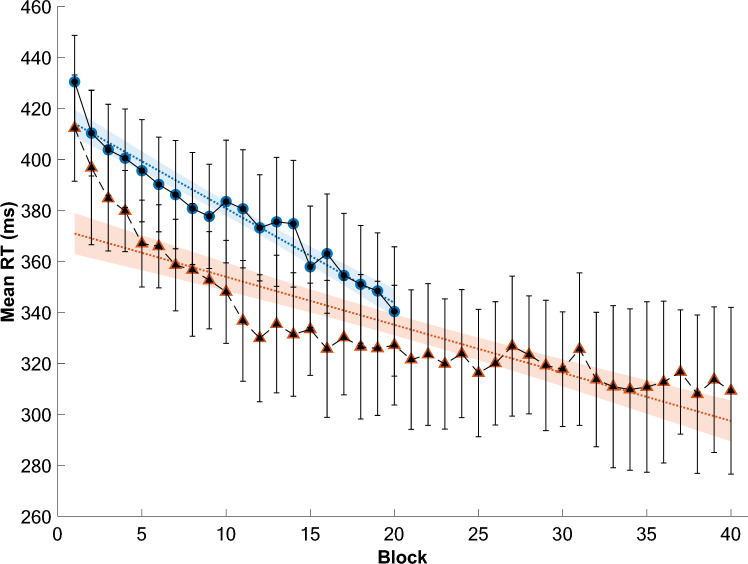
Evolution of mean response times (RT) across Blocks. Evolution of mean response times (RT) across Blocks by averaging within each Block all positions, all trials, and all monkeys in Experiment 1 (with sequences of 4-items, blue circles) and in Experiment 2 (with sequences of 5-items, orange triangles). Error bars represent 95% confidence intervals, dotted lines represent linear regressions fitted to each distribution and shaded areas represent predicted confidence intervals

General sequence learning was estimated by computing on each trial the average of RTs over the four positions in the sequence. For each participant, we averaged these mean RTs for each Block of 100 trials and Fig. 2 represents the evolution of mean RTs for the entire group of monkeys. These values were entered in a repeated measures one-way ANOVA with Block (1–20) as the within factor. The effect of Block was highly significant, *F*(19, 304) = 21.175, *p* < 0.001, *η*^2^ = 0.57. A linear regression also indicates that mean RTs decreased throughout the blocks of trials, *F*(1, 38) = 255, *p* < 0.001, Adjusted R^2^ = 0.93, (Block 1, *M* = 430.43, *SD* = 38.25; Block 20, *M* = 340.34, *SD* = 34.71), suggesting that monkeys learned the sequence.

**Fig. 3:**
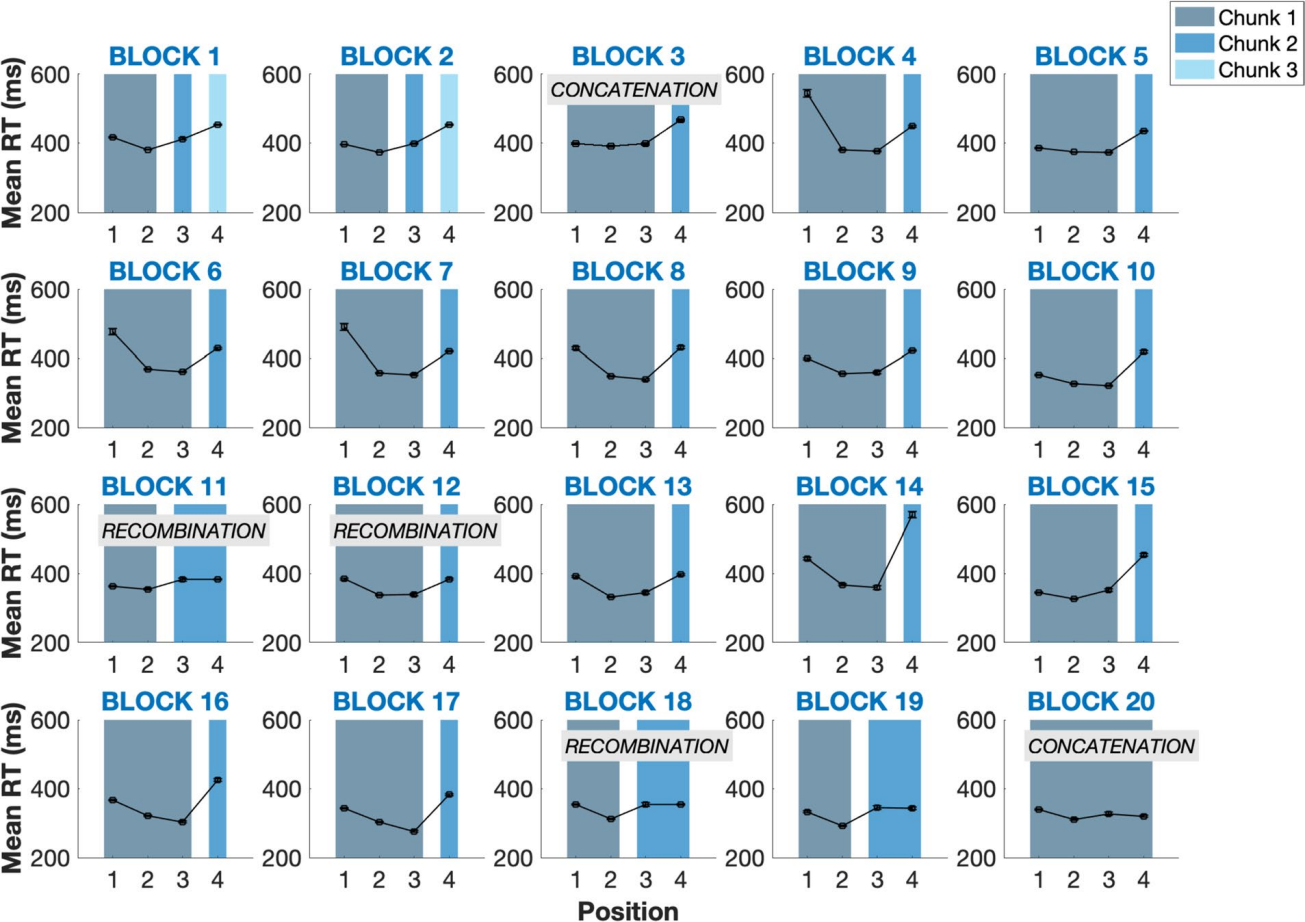
Evolution of the chunking pattern for one individual (Lips) throughout the task. Mean RTs per position across the 20 blocks of 100 trials for one baboon (Lips) showing the evolution of the chunking pattern. This individual initially parses the sequence into three chunks at the beginning of the task (i.e., Block 1) before concatenating the 3rd position with the first two into one chunk during Block 3. The sequence is recombined into two chunks of two positions at Block 11, before being recombined again in Block 12. The two chunks are again recombined during Block 18 and the sequence is fully concatenated into a single chunk during Block 20 (error bars represent 95% confidence intervals)

We adopted the same method as previously used for sequences composed of 9 positions (i.e., Tosatto et al. 2022) to study the chunking pattern of the sequence by monkeys. We considered successive positions *A* and *B* to be part of the same chunk as long as the transition time from one position to the next did not correspond to a significant increase in RT, otherwise an AB transition was supposed to mark a chunk boundary (Kennerley et al. 2004). Statistical significance was assessed through paired-sample t-tests for each pair of successive positions (significance threshold is set at 0.01 to correct for multiple comparisons^1^). Each time the RT of a pair's second position was significantly higher than the first position, it marked a chunk boundary. This analysis was applied on the mean RTs obtained at each position, for each Block of 100 trials and for each monkey (see Fig. 3 for an illustration of this procedure for one monkey).

With this method, we were able to quantify the number of chunks and their average size produced on each block by each monkey. Two linear regressions were conducted to test the effect of Block on the mean number of chunks and the mean chunk size respectively. These analyses revealed that the number of chunks significantly decreased across blocks (Block 1, *M* = 2.18, *SD* = 0.45; Block 20, *M* = 1.47, *SD* = 0.32; *F*(1, 18) = 98.6, *p* < 0.001, Adjusted R^2^ = 0.84) and that chunk size significantly increased across blocks (Block 1, *M* = 2.12, *SD* = 0.59; Block 20, *M* = 3.06, *SD* = 0.64; *F*(1, 18) = 83.4, *p* < 0.001, Adjusted R^2^ = 0.81). Note that we get exactly the same linear trends if we change the block size by taking 40 blocks of 50 trials or 10 blocks of 200 trials (see Supplementary Materials).

**Fig. 4:**
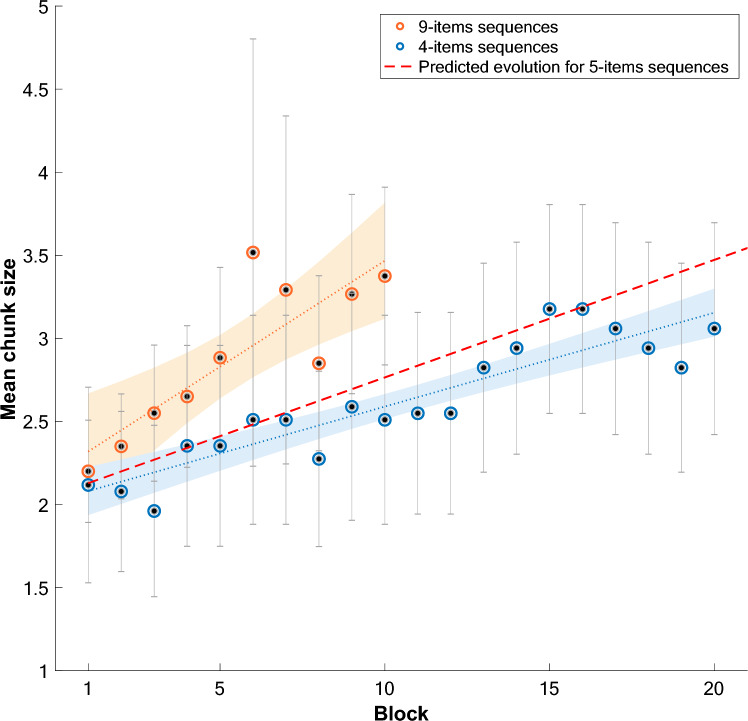
Evolution of chunk size for long (9-items in blue) and short (4-items in orange) sequences. The predicted evolution for 5-items sequences is given by the red dotted line. Mean chunk size (i.e., number of items per chunk) per block for long sequences (9-items, red dots) and short sequences (4-items, blue dots), error bars represent 95% confidence intervals. The red line illustrates the predicted mean chunk size per block for 5 items sequences, determined after Experiment 1 via a multiple regression with Block, Length and interaction as predictors. Dotted lines represent the same regression coefficients applied to 4 and 9-item sequences, shaded areas represent predicted confidence intervals

Additionally, for the average chunk size, we combined the present data on sequences of 4 items with the data collected in Tosatto et al. (2022) on sequences of 9 items to conduct a multiple regression analysis testing the effect of Block (1–20), Length (4 or 9) and the interaction of these two factors (see Fig. 4 for a representation of these data). This analysis revealed an effect of Block, Length and a significant interaction between these predictors (*F*(3, 26) = 34.8, *p* < *0.0*01, Adjusted R^2^ = 0.78). The individual predictors showed no main effect of Block (*t* = − 0.01, *p* = 0.98) and no main effect of Length (*t* = 1.04, *p* = 0.31), but a significant interaction indicating that the increase of chunk size across blocks differs between 4-items and 9-items sequences, (*t* = 3.12, *p* = 0.004).

Finally, we studied the two reorganization mechanisms reported in Tosatto et al. (2022). We found that 52.94% of the reorganizations were *concatenations* (which were observed in all monkeys) and 47.06% of the reorganizations were *recombinations* (which were observed in 15 monkeys). Table 1 provides the total number of concatenations and recombinations obtained for each block and for all monkeys in Experiment 1. A repeated measure ANOVA with Block and Mechanisms (concatenation vs. recombination) did not reveal any significant difference between these two reorganization mechanisms (all ps > 0.05).

**Table 1:** Total number of concatenations and recombinations per block for Experiment 1

Block	Concatenations	Recombinations
2	1	2
3	1	3
4	5	1
5	6	4
6	4	2
7	2	4
8	1	0
9	2	4
10	3	1
11	1	3
12	1	3
13	6	1
14	1	3
15	6	2
16	2	2
17	4	3
18	2	6
19	2	4
20	4	0
Total	54	48

### Discussion

Two main findings were obtained in the present study. First, we confirmed the results of a previous experiment led on the evolution of chunks while learning a 9-item visuo-motor sequence. As it was the case with a longer sequence, non-human primates learn 4-item sequences by segmenting the sequence into small chunks and, with extended practice, these chunks become longer and fewer. Second, this decrease in the number of chunks and this increase in chunks’ size is due to two types of reorganizations: the recombination of several preexisting chunks and the concatenation of two distinct chunks into one.

It is interesting to note that the final mean chunk size after producing the sequence 1000 times is only 2.51 (CI [1.88, 3.14]; *Min* = 1; *Max* = 4), and that after 2000 trials, it is still different from 4 (*Mean* = *3.06*; CI [2.42, 3.7]; *Min* = 1; *Max* = 4), indicating that baboons continue, on average, to segment this short sequence in several chunks. This is even more interesting considering that the mean chunk size for 9-item sequences was 3.38 (CI [2.85, 3.91]; *Min* = 1; *Max* = 8) after 1000 trials, indicating that chunk size varies with the length of the sequence. Chunking processes therefore seem to operate in interaction with the size of the sequence.

To further explore this interaction, we used the linear regressions presented in Fig. 4 to extrapolate the slope of the linear regression that should be obtained for a sequence length between 4 and 9. Indeed, it is possible to use regression models as predictive models as illustrated in Eq. (1):

1 $$y = \beta_{0} + \beta_{1} x_{1} + \beta_{2} x_{2} + \beta_{1.2} x_{1.2}$$

Here, the linear regression predicts the mean chunk size y as a function of the intercept β_0_, the slope coefficient β_1_ at block *x*_1_, the slope coefficient β_2_ for a sequence of length *x*_2_ and the interaction effect β_1.2_ between block and length. Using this formula, we can replace *x*_2_ by a constant C = 5 to model the predicted evolution of the mean chunk size across blocks for a sequence of 5 items. The resulting predictive line is represented on Fig. 4. According to this model, a mean chunk size greater than 4 (4.89) should be observed after 4000 trials for sequences of 5-items. This indicates that large chunks could only be formed after greater extended practice. Experiment 2 was designed to assess the predictive power of that model and test the hypothesis that the relationship between block, sequence length and chunk size is linear.

## Experiment 2

### Method

#### Participants

The same twenty-five Guinea baboons from the CNRS primate facility in Rousset (France) were tested in this study. For practical reasons, we stopped the experiment after 21 monkeys completed all scheduled trials (fifteen female and six males, age range 2.8–24.8 years). Sixteen out of these 21 baboons also performed Experiment 1.

#### Materials and methods

The apparatus, task, and stimuli were identical to those of Experiment 1. The only exception was sequence length, which was 5 items in Experiment 2. In order to avoid a familiarity effect with specific sequences in baboons, we designed 3 new sequences of 5 items, using the same method as in Experiment 1. Appendix 3 provides the sequences presented to each of the 21 baboons.

#### Procedure

Baboons were either presented with Sequence 1 (n = 7), Sequence 2 (n = 6) or Sequence 3 (n = 7) and had to produce it for 4000 successive trials. The number of trials was chosen in order to have baboons mastering the sequence to a point we had never tested, and which would seemingly allow them to form larger chunks, according to our predictions. RTs for each position of the sequence were recorded for all the trials. Experiment 2 occurred six months after Experiment 1 and to minimize the potential interference between the two experiments, baboons started this second experiment with series of random sequences.

### Results

On average, baboons required 8.5 days (*SD* = 3.36) to complete the 4000 trials, with a mean of 471.9 trials per day and a mean accuracy level of 92.7% (*SD* = 4.32). Incorrect trials were removed from the dataset (7.29%). RTs greater than 1000 ms were excluded and an additional recursive trimming procedure excluded RTs greater or smaller than 2.5 standard deviations from the subject’s mean for each of the five possible positions (18.02%). RTs for each of the five positions and for the 4000 trials were divided into 40 Blocks of 100 trials.

General sequence learning was estimated on mean RTs by a repeated measures one-way ANOVA with Block (1–40) as the within factor (see Fig. 2 for the evolution of mean RTs across Blocks).

The effect of Block was highly significant (Block 1, *M* = 412.27, *SD* = 18.7; Block 40, *M* = 309.24, *SD* = 42.02; *F*(39, 819) = 30.39, *p* < 0.001, *η*^2^ = 0.603). A linear regression also indicates that mean RTs decreased throughout the blocks of trials, *F*(1, 38) = 116, *p* < 0.001, Adjusted R^2^ = 0.75, (Block 1, *M* = 412,27 *SD* = 20.85; Block 20, *M* = 309.24, *SD* = 32.69), suggesting that monkeys learned the sequence.

A linear regression was conducted to test the effect of Block on the mean number of chunks. As in Experiment 1, this analysis revealed that the mean number of chunks significantly decreased across blocks, (Block 1, *M* = 2, *SD* = 0.32; Block 20, *M* = 1.52, *SD* = 0.39; *F*(1, 38) = 80.7, *p* < 0.001, Adjusted R^2^ = 0.67).

**Fig. 5:**
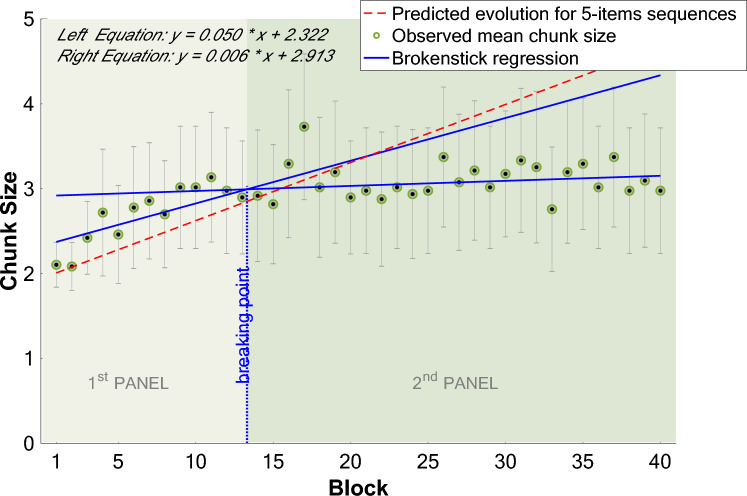
Evolution of the mean chunk size for 5-item sequences and the predicted evolution for 5-items sequences. Mean chunk size (i.e., number of items per chunk) per block for 5 items sequences, error bars represent 95% confidence intervals. Blue lines represent a broken stick regression separating data into 2 panels, at a calculated breaking point of 13.31. The red line illustrates the predicted mean chunk size per block for 5 items sequences, previously determined via a multiple regression

As for the average chunk size, Fig. 5 indicates that the distribution of values across blocks obeys two separate regimes: a first increase in chunk size that corresponds to the predicted line followed by a plateau. To account for these two regimes, we first used a broken stick linear regression (Quandt 1960) to determine the slope of the evolution before and after the plateau is reached. This analysis revealed an increase in chunk size during the first regime, with a slope coefficient of 0.05 (R^2^ = 0.67) and an almost flat slope of 0.006 (R^2^ = − 0.04) in the second regime, with a breakpoint (i.e., deceleration) after the 13th Block. A logistic growth model completes this analysis and indicates that the plateau is reached (i.e., the carrying capacity) at a size of 3.109 items (see Supplementary Fig. 5).

As for the reorganization mechanisms, we found that 55.6% of the reorganizations were *concatenations* (which were observed in all monkeys) and 44.4% of the reorganizations were *recombinations* (which were also observed in all monkeys). Table 2 provides the total number of concatenations and recombinations obtained for each block and for all monkeys in Experiment 2. A repeated measure ANOVA with Block and Mechanisms (concatenation vs. recombination) reveals a significant effect of Mechanism (*F*(1, 40) = 9.16, *p* = 0.007, *η*^2^ = 0.003) but no significant effect of Block or interaction (both ps > 0.05).

**Table 2:** Total number of concatenations and recombinations per block for Experiment 2

Block	Concatenations	Recombinations	Block	Concatenations	Recombinations
2	5	11	21	3	5
3	11	5	22	3	8
4	4	4	23	7	3
5	5	6	24	3	5
6	5	6	25	3	5
7	5	5	26	5	6
8	3	6	27	5	5
9	5	3	28	6	8
10	6	8	29	5	7
11	7	4	30	5	8
12	2	7	31	8	5
13	4	3	32	5	6
14	3	8	33	2	9
15	5	4	34	6	2
16	5	3	35	5	1
17	9	5	36	1	8
18	2	14	37	5	4
19	7	5	38	2	7
20	3	5	39	2	6
21	3	5	40	3	5
Total				180	225

### Discussion

Three main findings stem from Experiment 2. First, the results confirm the dynamics of the evolution of chunks observed on 4- (see Experiment 1) and 9-item (Tosatto et al. 2022) sequences: the sequence is initially parsed into small chunks and after extended practice, chunks become longer and fewer. Second, there seem to be a relationship between practice (i.e., repetitions of the sequence), sequence length and mean chunk size. In the current study, we were able to accurately predict the evolution of mean chunk size for a given sequence length using a linear regression model, with only three parameters: block, sequence length and the interaction between these factors. Third, by increasing the total number of trials (up to 4000), we found that the increase in chunk size progressively reached a plateau after 20 blocks of trials (the deceleration starting around Block 13), and the observed distribution of mean chunk size progressively deviated from the predicted distribution. This stabilization of the number of chunks suggests that there is a limit to chunk size that is related to sequence length and that the reorganization of chunking patterns stops after a large number of repetitions of the same sequence.

## General discussion

The present study was designed to assess the effect of sequence length on the evolution of chunks during sequence learning. The results from Experiment 1, conducted on 4-item sequences, confirmed the results obtained on 9-item sequences regarding the evolution of chunks. Indeed, even on smaller sequences, baboons initially segmented the sequence into small chunks of approximately 2 items. Throughout learning, chunks became fewer and longer via two mechanisms of reorganization of the chunking pattern: some chunks were simply grouped together (leading to a *concatenation*) while a new segmentation pattern arose between two previous chunks (leading to a *recombination*). Comparing the learning of 4- and 9-item sequences also revealed that the evolution of the mean chunk size varied as a function of sequence length. Experiment 2 replicated these findings with a sequence of 5-items. However, it also revealed that the linear increase in chunk size reached a plateau after extended practice suggesting that a limit was attained.

These results indicate that the role of practice on the evolution of chunks is important but limited. Indeed, after thousands of repetitions of the same sequence, the chunking pattern still evolved and some reorganizations were observed, but the mean chunk size itself reached a plateau (see Experiment 2). Additionally, after extended practice and contrary to what was expected, the sequence was not systematically produced in a single chunk of 4 (in Experiment 1) or 5 (in Experiment 2). These data clearly suggest that there is a functional limit to the maximum size of a chunk in sequence learning which is between 3 and 4.

Interestingly, as stated in the introduction, this functional limit or potentially optimal size of a chunk has been very consistently reported in the literature (Allen and Coyne 1988; Chase and Simon 1973; Cowan 2001; Johnson 1970). One important feature of the maximum chunk size is that it does not seem to depend on the paradigm used to study sequence learning. Indeed, in our case, one could argue that the SRT task performed by the baboons is quite simple, as they are trained successively on each item in the sequence and the visual sequence presented is overlapping the motor sequence of responses. These two features could influence the maximum chunk size reached. In other paradigms, such as the simultaneous chain paradigm (e.g., Terrace 1991; Terrace and Chen 1991a, b; Terrace et al. 2003), subjects are not trained sequentially and are presented simultaneously with all items in the sequence on a screen (e.g., photographs). Subjects therefore have to figure out the order in which each item must be selected by trial and error. Moreover, the spatial arrangement of all items changes at each trial, ensuring that subjects do not learn a single motor sequence but the order of selection of the items (e.g., bird → frog → flower). However, even with these considerable differences in paradigms, the typical reported chunk size is 3 to 4 items (Scarf et al. 2018; Terrace 2002). This indicates that this size is not a simple by-product of the fixed motor sequence or the sequential training used in our study but a more general feature of the memory system.

Note that the absolute chunk size may vary according to the processing applied to our data, and in particular whether or not a trimming procedure is used. Supplementary Fig. 2 shows that when data are not trimmed (Panel A), the average size of chunks is around 3.5, whereas for trimmed data (Panel B), this value is just above 3. For this reason, we can only assert that the maximum size will be between 3 and 4, but above all, in both cases, this size never exceeds 4 (on average). So, despite these variations, the conclusion remains that there is an intrinsic limit to the size of chunks in these implicit sequence learning processes.

On another hand, practice does not seem to be the only parameter on which chunk size depends. Indeed, our results indicate that sequence length also has an impact on chunking patterns. The initial mean chunk size for the three sequence lengths tested with our paradigm was always around 2 items, as evidenced by the intercepts of the regressions fitted to our distributions of chunk size (ɑ_1_ = 2.19; ɑ_2_ = 2.02; ɑ_3_ = 2.32, for the first panel). Differences in the chunk size distribution only arise throughout the task, as evidenced by the effect of sequence length and the different slopes of these regressions (β_1_ = 0.13; β_2_ = 0.56; β_3_ = 0.50, for the first panel). However, contrary to what we may expect, practice on longer sequences generates larger mean chunk sizes compared to shorter sequences. Indeed, after 1000 repetitions of the same sequence, the mean chunk size for sequences of 9-items is 3.38, it is 3.02 for 5-item sequences and 2.39 for 4-item sequences. Although we were expecting short sequences of 4 items to generate chunk sizes close to 4, we observed the reverse: maximum mean chunk sizes were obtained for longer sequences.

It is important to note that, here, we studied the chunking pattern by comparing, in each block, the mean RT of pairs of successive items. This method has been widely used in the literature (e.g., Bera et al. 2021; Diedrichsen and Kornysheva 2015; Kennerley et al. 2004) and appears as efficient and reliable as other methods such as the non-parametric algorithm proposed by Alamia et al. (2016). However, as there exists no consensus method to identify chunks, this method, like all others, relies on somewhat arbitrary choices. Thus, different methods could lead to identifying slightly different chunking patterns (Alamia et al. 2016; Gilchrist 2015) and we need to be cautious about drawing strong conclusions regarding the specific chunking patterns identified here. For example, we applied the method used by Scarf et al. (2018) to identify chunk boundaries to the untrimmed data collected in Experiment 1. This method proposes to locate, for each trial, the peak RT(s) preceded and followed by shorter RTs to identify the start of a chunk. This allows to identify the different chunking patterns used during the task on each trial. Supplementary Fig. 4, panel A, illustrates this reasoning applied to our data for one baboon (Angele) during Block 1. We identified the use of several chunking patterns during this block, but for the majority of trials Angele adopted a parsing of the 4-item sequence in two chunks of two items. Using the same reasoning for each block and each baboon, we then calculated the mean chunk size per block throughout the task (Supplementary Fig. 4, panel B). This revealed similar results to those obtained initially, with a significant increase of chunk size across blocks, albeit several chunking patterns were identified at each block and the calculated mean chunk sizes are therefore slightly smaller than the ones we found with our method. Additionally, we found that our results do not change when modifying the fine tuning of our analysis such as block size (see Supplementary Materials), allowing us to be confident on the general trends observed in the present study.

Therefore, it seems that sequence length partly constraints the mean chunk size and its evolution. Chunking, as a mechanism of compression of information in memory, seems to depend on the amount of information that needs to be compressed. In this regard, when the length of the practiced sequence increases, more information needs to be stored in a few chunks, resulting in overall bigger chunks. By contrast, when the practiced sequence is very short, there seems to be no advantage to form a single long chunk, and two smaller chunks looks more economical in terms of cognitive resources.

These results also raise the question of the memory system involved in chunking mechanisms. Indeed, chunking is typically viewed as a process used to compress information because of limits of storage in working or short-term memory. However, our studies, among many other sequence learning studies, observe chunking patterns across hundreds of repetitions of the same sequence. Therefore, it seems impossible that short term memory is the only memory system involved in chunking mechanisms. This is further indicated by the fact that, with extended training, we observed a performance plateau reached in the chunk size (in Experiment 2). A possible interpretation here is the switching from a learning phase—possibly involving primarily short-term memory—and an execution phase after the sequence is learned, related to long-term memory. Regarding this issue, Terrace (2002) suggested to distinguish between input chunks, i.e., the parsing used to encode the input in short-term memory when faced with storage constraints, and output chunks, i.e., how the performance is impacted by retrieving chunks in long-term memory and « uploading» the adequate response program. In this view, chunk boundaries as evidenced by the temporal pauses we observe in the performance would reflect the uploading time. This metaphor of the brain executing sequences as a computer and retrieving response programs has been widely supported in the field of motor sequence learning (Abrahamse et al. 2013; Verwey 2001; Wymbs et al. 2012).

However, these theoretical accounts are questionable, especially in terms of biological plausibility. Recently, new propositions have argued that associative, or Hebbian learning principles could constitute a simpler and more realistic account of sequence learning (e.g., Endress and Johnson 2021; Tovar et al. 2018; Tovar and Westermann 2023). For instance, when learning sequences of 3 items (e.g., A–B–C), both baboons and humans display greater decrease in RT on the third item of the sequence compared to the second (i.e., A > B > C; Minier et al. 2016; Rey et al. 2019). The authors have suggested that the item C may have benefited from an association not only between B and C, but also from an association between A and C. This could account for the formation of a chunk ABC based on associative principles. Indeed, Tovar et al. (2018) have since implemented a computational model where the strength of connection between neurons coding for items is calculated with a Hebbian algorithm and have shown that presenting the patterns A–B and B–C repeatedly allows their model to create and strengthen associations between A and B and B and C but also between A and C. Hebbian learning models can therefore account for the creation of a chunk. However, these types of models are also limited as they do not account for segmenting a longer input in distinct chunks yet, not to mention the segmentation of the input according to its length. Future computational studies should make it possible to solve this problem of segmenting long sequences into chunks, based on the Hebbian principle or by taking into account other principles, such as the notion of pair-coding neurons (see Rey et al. 2022).

Nevertheless, it is interesting to note that taking into account chunk length as a pragmatic characteristic of the chunking mechanisms is not implemented in any computational models of chunking. For example, in the chunking model PARSER (Perruchet and Vinter 1998), chunks can progressively grow larger by concatenating smaller chunks. But the growth of chunks and the final mean chunk size does not depend on the length of the initial pattern, nor does it stabilize with a great number of repetitions. The present data therefore provide new and challenging empirical evidence for current computational models of sequence learning, associative learning and chunking.

## Electronic supplementary material

Below is the link to the electronic supplementary material.

Supplementary file1 (PDF 813 KB)

## Data Availability

Data from the experiment are available on Open Science Framework at https://osf.io/gjvtn/?view_only=dabacf279dd54ff8ac47a2f992896b85.

## Appendix 1

Mean response times over the entire group of baboons for each of the 72 possible transitions calculated from the 1000 random trials.

**Table:**

1st position in transition	2nd position in transition								
	1	2	3	4	5	6	7	8	9
1	–	519	573	495	482	509	521	497	543
2	569	–	553	513	474	511	523	491	509
3	558	519	–	513	472	488	544	493	512
4	551	517	560	–	464	509	522	482	546
5	549	504	552	501	–	483	535	479	527
6	567	515	546	507	484	–	533	483	511
7	555	504	558	475	463	516	–	484	541
8	554	512	540	485	448	472	512	–	507
9	546	512	540	514	460	464	550	485	–

All transitions are in milliseconds (ms) and correspond to the time elapsed between the disappearance of the red circle from the 1st position of the Transition and the monkey’s touch on the 2nd position of the Transition. For example, consider the transition [4–8] from Position 4–8 (4 being the first position of the transition and 8 being the second position). When the red circle was on Position 4, baboons touched it and the target moved to Position 8. The mean response times for that transition [4–8], i.e., 482 ms, corresponds to the time baboons took on average to move from Position 4 to Position 8 (i.e., from the baboon’s touch on Position 4 to the baboon’s touch on Position 8).

## Appendix 2

List of the sequences used in Experiment 1 and corresponding mean transition times.

**Table:**

Sequence	Position				Mean transition time (ms)			Mean	SD
	1	2	3	4	T1	T2	T3		
1 (N = 4)	1	5	6	8	482	483	483	483	0.91
2 (N = 5)	5	6	8	4	483	483	485	483	1.1
3 (N = 3)	6	9	4	2	511	514	517	514	3.1
4 (N = 5)	3	4	2	7	513	517	523	518	5.3

## Appendix 3

List of the sequences used in Experiment 2 and corresponding mean transition times.

**Table:**

Sequence	Position					Mean transition time (ms)				Mean	SD
	1	2	3	4	5	T1	T2	T3	T4		
1 (N = 8)	1	4	6	9	2	495	509	511	512	507	7.9
2 (N = 6)	2	8	9	4	7	491	507	514	522	509	13.2
3 (N = 7)	3	5	2	9	4	472	504	509	514	500	19

## Footnotes

1 In the case of a large number of comparisons (8 in our case), there is a debate over the fact that Bonferroni and Holm corrections may overcorrect and set a significance threshold unnecessarily low, which increases the risk of type II error (e.g., Lee and Lee 2018). We have therefore decided to adjust our alpha threshold to the stricter conventional threshold of 0.01, considering that only a very significant difference between mean RTs would mark a chunk boundary.
