# Supplementary material for: Chunking as a function of sequence length
Source: Anim Cogn. 2024 Mar 2;28(1):2. doi: 10.1007/s10071-024-01835-z (PMC11671558; doi:10.1007/s10071-024-01835-z)
Supplement: Supplementary file 1 — Supplementary file1 (PDF 813 KB) [file 10071_2024_1835_MOESM1_ESM.pdf]

Supplementary Materials

Supplementary Methods

1. *Sequence design*

As stated in the main text, we designed our sequences so that each transition time (TT) between pairs of items would be shorter than the next one (TT1≤TT2≤TT3≤TT4). This was done so that a decreasing RT between successive responses would reflect anticipation, and thus learning, and not a shorter distance between targets. However, considering that our method to identify chunks consists in tracking longer response times between successive responses, we carefully selected our sequences so that the increasing TT between items would be as low as possible. In both experiments, the maximum average difference between TTs in the same sequence was 5 ms (see Appendix A). In fact, we ensured that the difference between TTs belonging to the same sequence was not significant, by conducting a series of one-way ANOVAs with the transitions used in each sequence as a factor (all $p>.05$). Appendixes B and C (in the main text) provide the details of all the sequences we used, i.e., the sequence itself, the average response times for each transition and the number of monkeys that saw each sequence.

2. *Trimming procedure*

Working with non-human primates that perform a self-paced task and are semi-free in an outdoor enclosure, the data we collect can be noisy compared to data collected in humans performing the same task. For instance, with baboons, on some occasions, the touch screen does not detect their first touch because their hands are dirty and they have to produce a second touch to receive the reward. This produces longer RTs and a bimodal distribution as illustrated in the Supplementary Figure 1: the distribution of all RTs before trimming from Experiment 1 are centered around 500ms, followed by longer RTs constituting a second distribution centered around 1000ms. This led us to delete a large number of observations in both Experiment 1 and

2. This is to ensure that, as much as possible, the RTs processed in our results are related to the execution of the sequence, and not a system failure to record the first touch. Using this trimming procedure also ensures that the data processing for Experiment 1 and 2 is similar to that performed in Tosatto et al. (2022), the results of which are compared with those of Experiments 1 and 2.

However, to alleviate any concerns related to data trimming and its impact on our results we provide a comparison of the evolution of the number of chunks and chunk size for trimmed and untrimmed data for Experiment 1 (see Supplementary Figure 2). For both untrimmed and trimmed data, the trend of our results does not change and the linear regressions fitted to these data are very similar (see Supplementary Table 1), indicating that data trimming does not determine the shape of our results and conclusions.

3. Block segmentation

We chose to segment each baboon's performance in blocks of 100 trials for both Experiment 1 and 2. This segmentation is somewhat arbitrary but is justified by several factors. First, as our goal was to compare the present study to another experiment led on 9-item sequences (Tosatto et al., 2022). We thus considered parsing our task in a similar way, using equivalent blocks of 100 trials.

Furthermore, we needed to have enough trials per position and per monkey to obtain sufficiently robust averages in order to carry out the calculation that allows us to delimit the chunks. By having blocks composed of too few trials, the risk is to have a higher variability and a less robust average per position and per monkey. One-hundred trials per block ensures that, after data trimming, even if we exclude up to 25% of the trials, our calculation will include at least 75 trials, guaranteeing very robust averages per item and monkey.

On another hand, one might think that 100 trials is a large window during which many changes in the chunking pattern might occur, rendering our analysis too superficial and unable

to account for fast evolutions of the chunking pattern. To assess the effect of the segmentation on the results in our two experiments, we compared the evolution of the number of chunks and chunk size obtained when segmenting both tasks in blocks of 200 and 50 trials (see Supplementary Figure 3). As evidenced by the robust general trend of a decrease in the number of chunks and an increase of chunk size regardless of the number of trials per block, the block segmentation of 100 trials per block does not impact our result in a considerable way. This is also confirmed by the very similar regression coefficients obtained when fitting linear models to these different distributions (see Supplementary Tables 2).

Supplementary Figure 1

Distribution of raw RTs for Experiment 1

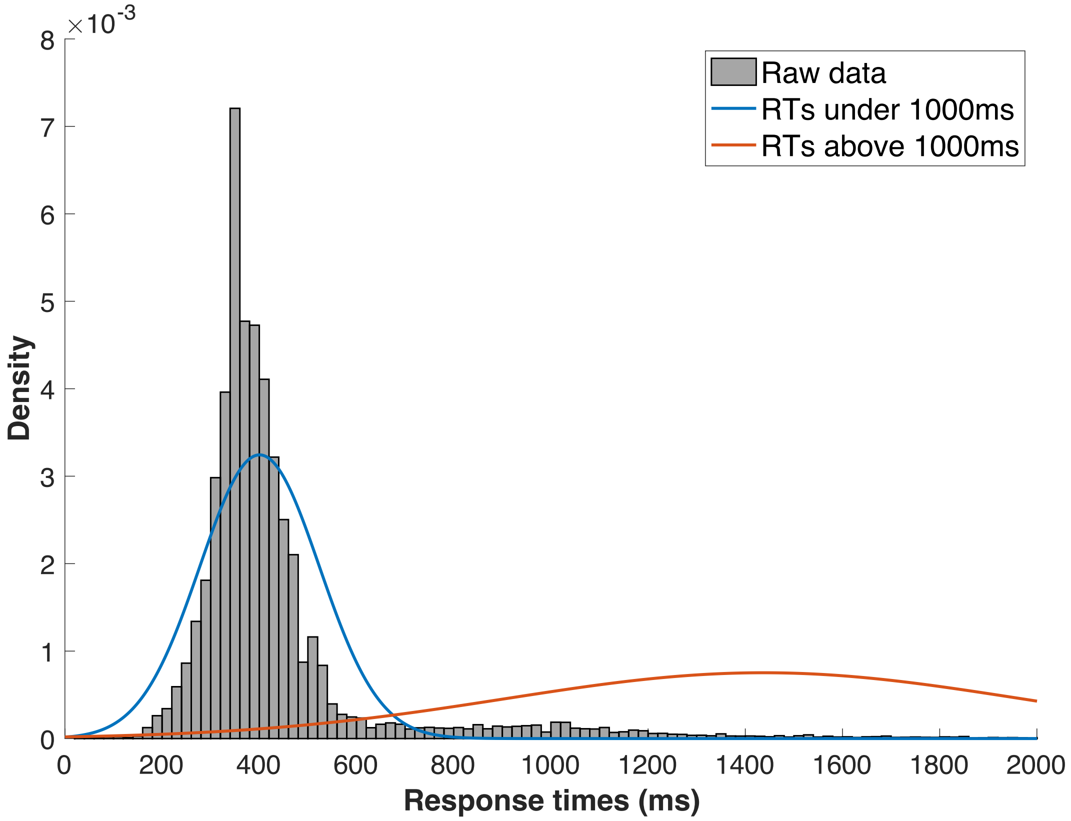

Note. Density plot of raw RTs (before trimming) in Experiment 1, showing a normal function fitted to a distribution of RTs shorter than our trimming cut-off of (1,000ms) in blue and a normal function fitted to a distribution of RTs longer than the cut-off in red.

Supplementary Figure 2

Evolution of mean number of chunks and mean chunk size per block without (A) and with data trimming (B) for Experiment 1

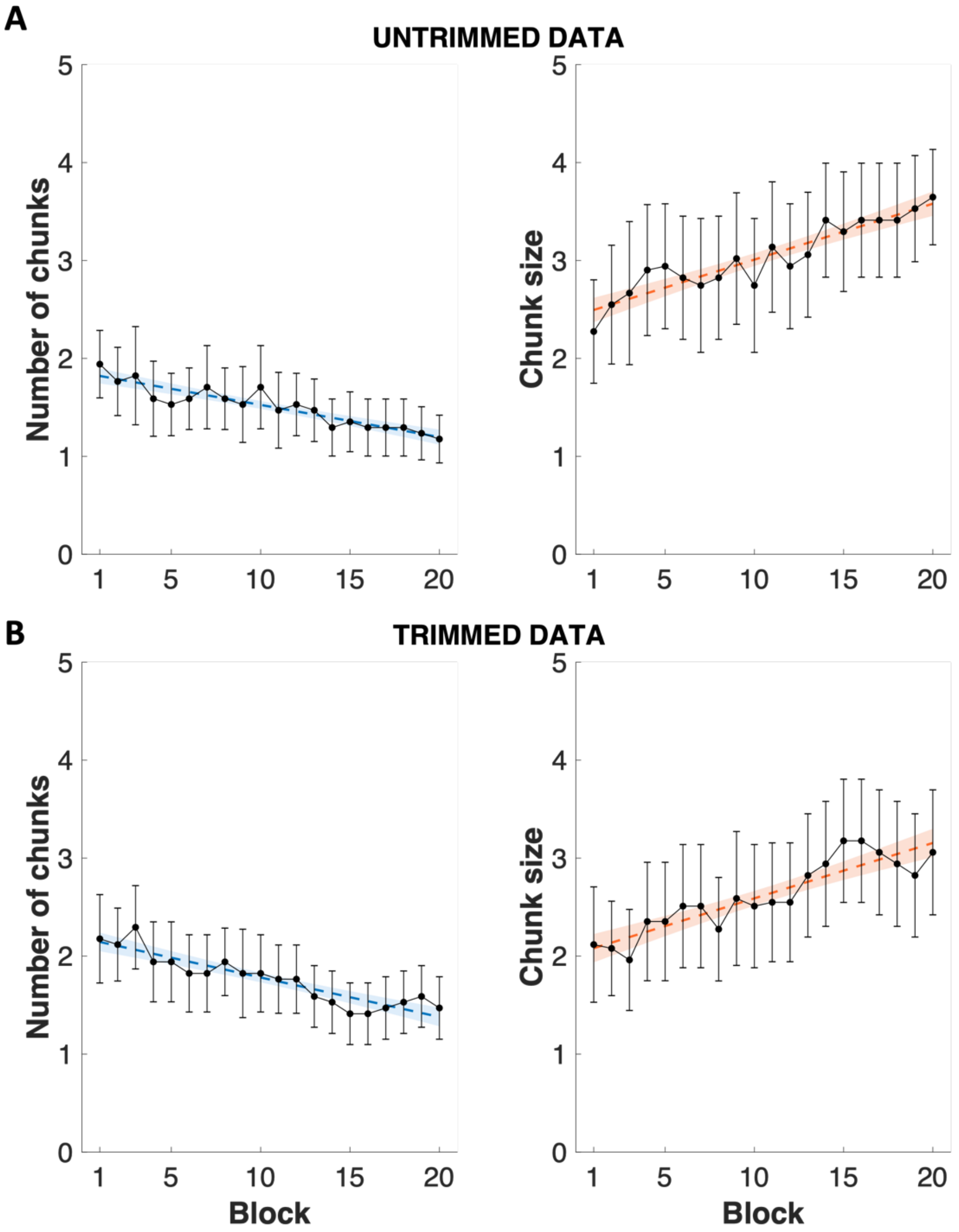

Note. Only errors were removed in the analysis for A. The trimming procedure does not significantly affect the result pattern (see Supplementary Table 1).

Supplementary Figure 3

Evolution of mean number of chunks and mean chunk size when performance is segmented in 10 blocks of 200 trials (A) and 40 blocks of 50 trials (B)

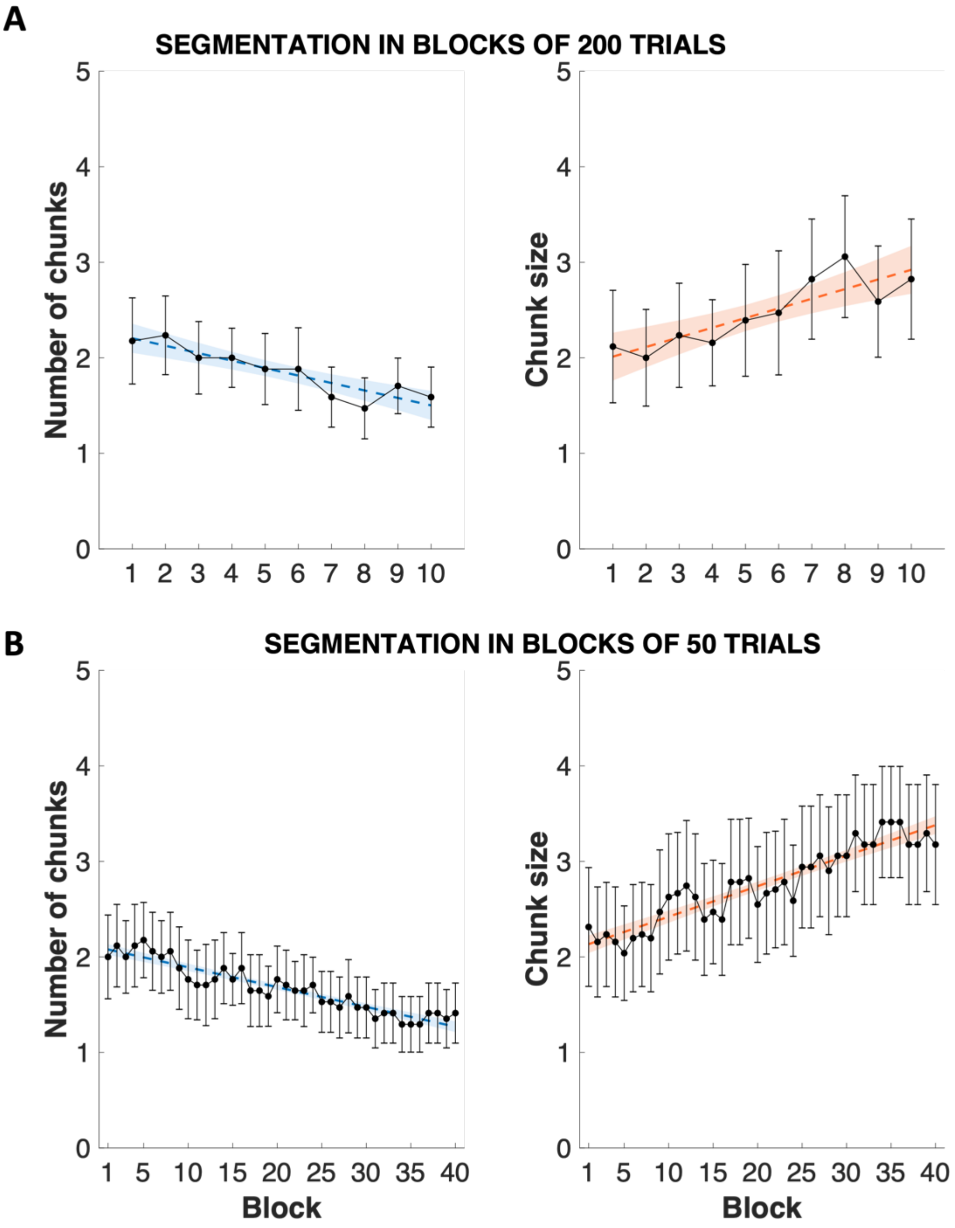

Note. The block segmentation does not significantly affect the result pattern (see Supplementary Table 2).

Supplementary Figure 4

Illustration of the chunk identification method from Scarf et al. (2018)

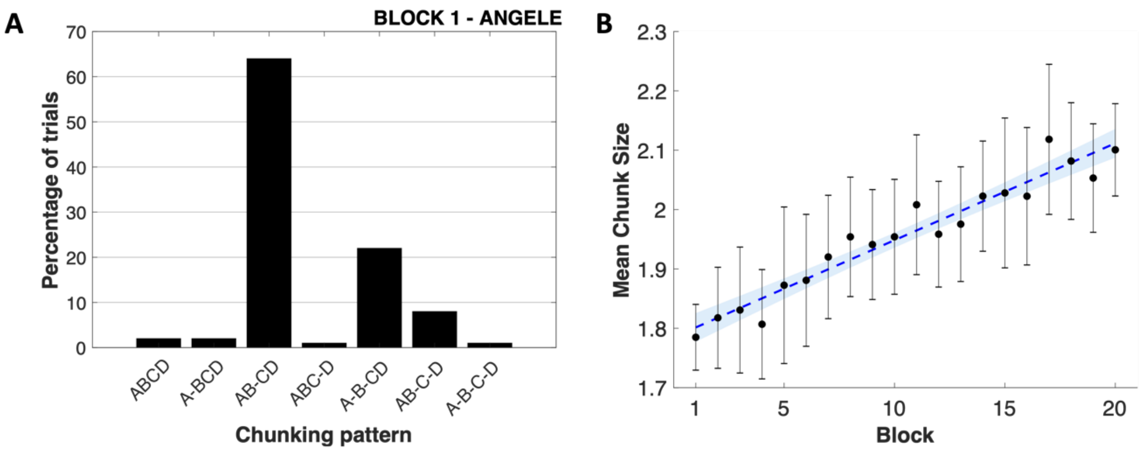

Note. A. Illustration of the application of the trial-by-trial method for one baboon (Angele) for Block 1. B. Mean chunk size at the group level in Experiment 1 using the chunk identification method from Scarf et al. (2018). Errorbars represent 95% confidence intervals, dotted blue line represents a fitted linear regression ($F(1,19)$=250, p<.001, Adjusted R^2=.93) and shaded areas represent predicted confidence intervals.

Supplementary Figure 5

Mean chunk size in Experiment 2, fitted logistic growth and broken stick regression models

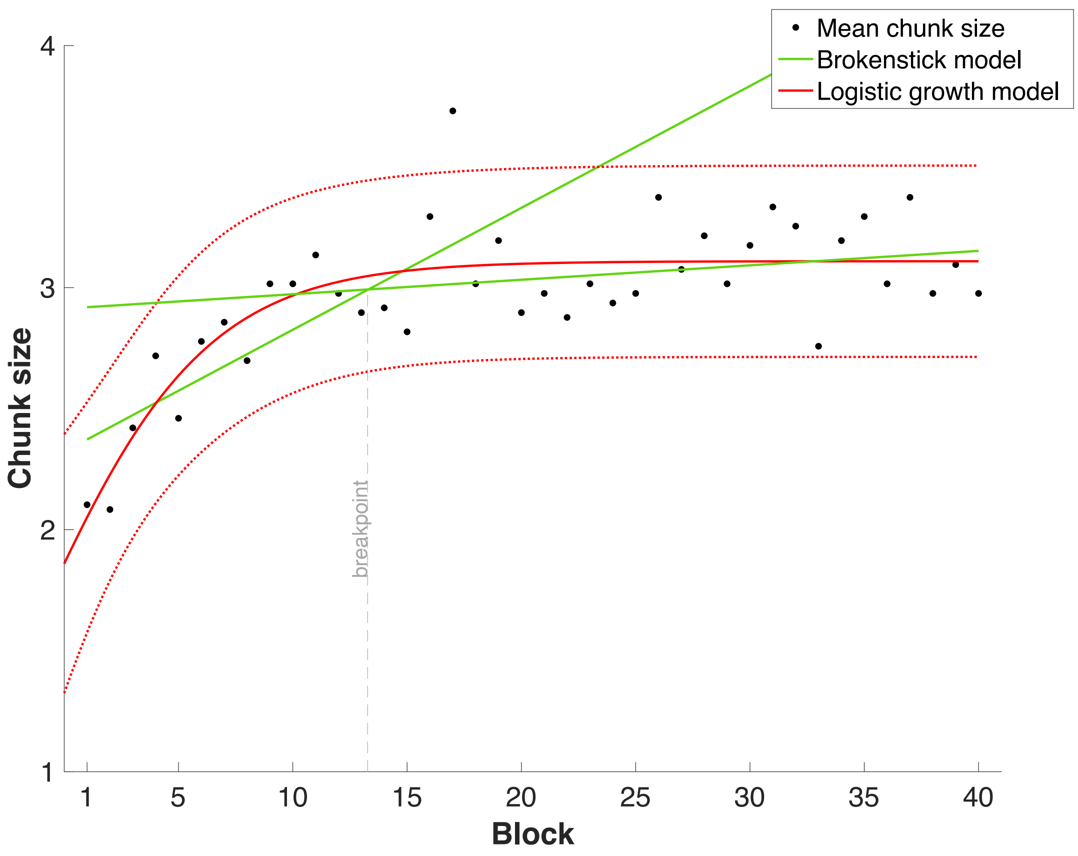

Note. Both model options show that chunk size stabilizes after several blocks of trials. The broken stick regression model (in green) informs about the breakpoint at which chunk size stabilizes, whereas the logistic growth model (in red, doted lines indicate 95% confidence intervals) indicates the carrying capacity, i.e., maximum chunk size.

Supplementary Table 1

Results of linear regressions led on the mean number of chunks and chunk size per block, for both trimmed and untrimmed data in Experiment 1

Untrimmed data

Number of chunks – Trimmed data

	Estimate	SE	t stat	p value
Intercept	1.43	.03	49.47	< .001
Block	-.007	.001	-5.84	< .001

Number of observations: 40, Error degrees of freedom: 38
Root Mean Squared Error: 0.0895
R-squared: 0.473, Adjusted R-Squared 0.459
F-statistic vs. constant model: 34.1, p-value = 9.57e-07

Chunk size – Trimmed data

	Estimate	SE	t stat	p value
Intercept	3.7	.08	45.36	< .001
Block	.02	.003	5.23	< .001

Number of observations: 40, Error degrees of freedom: 38
Root Mean Squared Error: 0.253
R-squared: 0.419, Adjusted R-Squared 0.404
F-statistic vs. constant model: 27.4, p-value = 6.4e-06

Trimmed data

Number of chunks – Trimmed data

	Estimate	SE	t stat	p value
Intercept	1.96	.035	56.29	< .001
Block	- .013	.001	- 8.98	< .001

Number of observations: 40, Error degrees of freedom: 38
Root Mean Squared Error: 0.108
R-squared: 0.68, Adjusted R-Squared 0.671
F-statistic vs. constant model: 80.7, p-value = 6.19e-11

Chunk size – Trimmed data

	Estimate	SE	t stat	p value
Intercept	2.64	.07	31.49	< .001
Block	.02	.004	4.54	< .001

Number of observations: 40, Error degrees of freedom: 38
Root Mean Squared Error: 0.26
R-squared: 0.352, Adjusted R-Squared 0.335
F-statistic vs. constant model: 20.6, p-value = 5.53e-05

Supplementary Table 2

Results of linear regressions led on the mean number of chunks and chunk size per block, for block sizes of 200 trials and 50 trials in Experiment 1

Blocks of 200 trials

Number of chunks

	Estimate	SE	t stat	p value
Intercept	2.3	.08	29.75	< .001
Block	-.08	.012	-6.31	< .001

Number of observations: 10, Error degrees of freedom: 8
Root Mean Squared Error: 0.112
R-squared: 0.833, Adjusted R-Squared 0.812
F-statistic vs. constant model: 39.9, p-value = 0.000229

Chunk size

	Estimate	SE	t stat	p value
Intercept	1.9	.13	15.14	< .001
Block	.1	.02	4.97	.001

Number of observations: 10, Error degrees of freedom: 8
Root Mean Squared Error: 0.185
R-squared: 0.755, Adjusted R-Squared 0.724
F-statistic vs. constant model: 24.7, p-value = 0.0011

Blocks of 50 trials

Number of chunks

	Estimate	SE	t stat	p value
Intercept	2.1	.03	72.96	< .001
Block	-.02	.001	-16.96	< .001

Number of observations: 40, Error degrees of freedom: 38
Root Mean Squared Error: 0.0893
R-squared: 0.883, Adjusted R-Squared 0.88
F-statistic vs. constant model: 288, p-value = 2.55e-19

Chunk size

	Estimate	SE	t stat	p value
Intercept	2.1	.04	44.2	< .001
Block	.03	.002	15.81	< .001

Number of observations: 40, Error degrees of freedom: 38
Root Mean Squared Error: 0.148
R-squared: 0.868, Adjusted R-Squared 0.865
F-statistic vs. constant model: 250, p-value = 2.65e-18
